# 2-Methacryloyloxyethyl Phosphorylcholine Polymer Coating Inhibits Bacterial Adhesion and Biofilm Formation on a Suture: An *In Vitro* and *In Vivo* Study

**DOI:** 10.1155/2020/5639651

**Published:** 2020-10-01

**Authors:** Taizo Kaneko, Taku Saito, Takeo Shobuike, Hiroshi Miyamoto, Junpei Matsuda, Kyoko Fukazawa, Kazuhiko Ishihara, Sakae Tanaka, Toru Moro

**Affiliations:** ^1^Sensory & Motor System Medicine, Graduate School of Medicine, The University of Tokyo, 7-3-1 Hongo, Bunkyo-ku, Tokyo 113-8655, Japan; ^2^Sensory Department of Pathology and Microbiology, Faculty of Medicine, Saga University, Saga 849-8501, Japan; ^3^Project Management Department, KEISEI Medical Industrial Corporation Limited, 3-19-6, Hongo, Bunkyo-ku, Tokyo 113-0033, Japan; ^4^Department of Materials Engineering, School of Engineering, The University of Tokyo, 7-3-1 Hongo, Bunkyo-ku, Tokyo 113-8656, Japan; ^5^Division of Science for Joint Reconstruction, Graduate School of Medicine, The University of Tokyo, 7-3-1 Hongo, Bunkyo-ku, Tokyo 113-8655, Japan

## Abstract

Initial bacterial adhesion to medical devices and subsequent biofilm formation are known as the leading causes of surgical site infection (SSI). Therefore, inhibition of bacterial adhesion and biofilm formation on the surface of medical devices can reduce the risk of SSIs. In this study, a highly hydrophilic, antibiofouling surface was prepared by coating the bioabsorbable suture surface with poly(2-methacryloyloxyethyl phosphorylcholine (MPC)-co-n-butyl methacrylate) (PMB). The PMB-coated and noncoated sutures exhibited similar mechanical strength and surface morphology. The effectiveness of the PMB coating on the suture to suppress adhesion and biofilm formation of methicillin-resistant *Staphylococcus aureus* and methicillin-susceptible *Staphylococcus aureus* was investigated both *in vitro* and *in vivo*. The bacterial adhesion test revealed that PMB coating significantly reduced the number of adherent bacteria, with no difference in the number of planktonic bacteria. Moreover, fluorescence microscopy and scanning electron microscopy observations of adherent bacteria on the suture surface after contact with bacterial suspension confirmed PMB coating-mediated inhibition of biofilm formation. Additionally, we found that the PMB-coated sutures exhibited significant antibiofouling effects *in vivo*. In conclusion, PMB-coated sutures demonstrated bacteriostatic effects associated with a highly hydrophilic, antibiofouling surface and inhibited bacterial adhesion and biofilm formation. Therefore, PMB-coated sutures could be a new alternative to reduce the risk of SSIs.

## 1. Introduction

Surgical site infections (SSIs) are the most common surgical complications, accounting for approximately 40% of all healthcare-associated infections [1] and 5% of all surgical complications [2]. SSIs result in further surgical intervention, prolonged treatment period, and unexpected high medical costs [3, 4]. Several factors are believed to be involved in the onset of SSIs, including implanted surgical sutures [5].

It is widely accepted that initial bacterial adhesion to biomaterial surfaces and biofilm formation secondary to bacterial adhesion are primary causes of SSI [6, 7]. Once a biofilm has formed, it protects bacteria from the host's immune system as well as systemically and locally applied antibiotics [7], thus rendering the SSI intractable [4, 7]. Therefore, inhibition of the initial bacterial adherence and biofilm formation on the surface of implanted sutures could be an effective countermeasure to reduce the incidence of SSIs.

Although antimicrobial agent-coated (e.g., triclosan [8]) suture surfaces reportedly suppress bacterial adhesion and biofilm formation, these antibacterial agents exert potential toxicity and side effects, including cytotoxicity, toxic side products, and antibiotic resistance [9, 10]. Due to these defects, the development of alternative suture materials with adequate antibiofouling ability and excellent biocompatibility has clinical value.

Several polymers can be used for surface modification of medical devices to prevent undesired biological responses [11–14]. Among them, zwitterionic phosphorylcholine-based polymers are the most attractive owing to their wide applicability to modify medical devices [15, 16]. For instance, 2-methacryloyloxyethyl phosphorylcholine (MPC) polymers are commonly used to provide highly hydrophilic and biocompatible surfaces [17–20]. Many medical devices, such as intravascular stents, ventricular assist devices, contact lenses, and hip acetabular liners, have been developed using MPC polymers and are clinically widespread [20–24]. Besides, the medical applicability and safety of MPC polymers are well established [20]. Thus, we expected that MPC polymer coating could be an ideal surface modification material with potential secondary benefits related to the inhibition of bacterial infection on the suture surface [25]. Poly(MPC-co-n-butyl methacrylate) (PMB) is one of the MPC polymers most widely used for surface modification of medical devices. Therefore, this study aimed to investigate the effects of PMB-coated sutures on initial bacterial adhesion and subsequent biofilm formation, both *in vitro* and *in vivo*.

## 2. Materials and Methods

### 2.1. Materials

The MPC polymer, PMB (Lipidure®-CM5206, NOF, Tokyo, Japan) and absorbable polyglactin sutures (KRAYON Plus®, KEISEI Medical Industrial Ltd., Tokyo, Japan) were used in this study. Other reagents, solvents, and analysis kits are commercially available and were used as obtained.

### 2.2. Surface Coating of Sutures with PMB

PMB was dissolved in 100 mL ethanol at a concentration of 1.0 wt%. The suture was immersed in the solution for several minutes using a dedicated coating machine and thoroughly air-dried for 10 min, followed by drying for 30 min at 50°C in a hot air oven. The dried suture was cut to 10 mm in length and wrapped in aluminum pouches for sterilization, respectively. All prepared sutures were sterilized with ethylene oxide gas under sterilization conditions according to ISO 11135:2014, prior to the experiment.

### 2.3. Characterization of Coated Sutures

#### 2.3.1. Confirmation of the Presence of PMB Coating by Various Methods

The surface of PMB-coated sutures was examined by X-ray photoelectron spectroscopy (XPS) (AXIS-HSi, Shimadzu/KRATOS, Kyoto, Japan) using Mg K*_α_*sources. High-resolution scans for C_1s_ and P_2p_ were acquired at a photoelectron takeoff angle of 90°. The energies of all spectra were corrected using the C_1s_ energy calibration peak at 285.0 eV. We visualized the phosphorylcholine groups on PMB's side chains by rhodamine 6G staining (Wako Pure Chemical Industries, Ltd, Tokyo, Japan) [19]. All samples were immersed in the rhodamine 6G solution for 30 s, washed twice with distilled water for 30 s, and let dry at room temperature. After staining, suture samples were observed under a fluorescence microscope (BZ-X700; Keyence, Japan). Besides, the absolute amount of phosphorus atoms on suture surfaces was measured by the molybdenum method [26].

#### 2.3.2. Mechanical Property Measurement

The diameter and weight of noncoated and coated sutures were measured at five different positions along the length of the suture. The average diameter was calculated using an electronic thickness gauge and expressed as millimeter. The weight was determined using a precision electronic balance and expressed as milligram. The tensile strength of coated and noncoated sutures was assessed by straight-pull tests, using an Instron 5960 Universal testing system (Instron, Canton, MA, USA) under the procedures outlined in the United States Pharmacopeia standard. Ten specimens were tested, and the results are expressed as mean ± standard deviation.

#### 2.3.3. Contact Angle Measurement

Static water contact angle measurements on the suture surface were carried out by the half-angle method using a goniometer (Model CA-S Micro2; Kyowa Interface Science Co., Ltd., Saitama, Japan). In brief, the suture attached to the measurement device jig was sprayed with ultrapure water, and the water contact angles were directly measured after 1.0 min using a contact angle meter. Fifteen specimens were tested, and the results are expressed as mean ± standard deviation.

### 2.4. Bacterial Strain Culture

Two different types of gram-positive bacteria strains were used in this study, including methicillin-resistant *Staphylococcus aureus* (MRSA) UOEH6 (University of Occupational and Environmental Health Hospital, Fukuoka, Japan), which was isolated from blood samples of a septic patient, and *S. aureus* (MSSA) NBRC12732 (Biological Resource Center, National Institute of Technology and Evaluation, Chiba, Japan). They were stored at -80°C in a glycerol stock. Bacteria were cultured overnight in Tryptic Soy Broth (TSB; Eiken Chemical, Tokyo, Japan) at 37°C. After incubation, bacterial cells were harvested by centrifugation and resuspended in PBS at a density of 5.0 × 10^8^ bacteria/mL.

### 2.5. Evaluation of Bacterial Adhesion

To allow adhesion to the suture samples, the suture samples were exposed to 5.0 × 10^8^ bacterial cells in 24-well plates and incubated for 1.0 h at 37°C. Subsequently, suture samples were placed in 1.5 mL tubes filled with 1.0 mL PBS, followed by vortexing and ultrasonication to scrape off firmly adhered bacteria from suture surfaces. The solutions were collected after ultrasonication, then serially diluted 10-folds, and plated on lysogeny broth plates supplemented with 7.5% sodium chloride. The CFU numbers for adherent and planktonic bacteria were counted after incubation at 37°C for 1 day. For nucleic acid staining in bacteria, the suture samples were stained with SYTO® 9 green fluorescent nucleic acid stain (FilmTracer™ LIVE/DEAD Biofilm Viability kit; Molecular Probes, Eugene, OR, USA) at room temperature for 30 min. After staining, suture samples were observed under a fluorescence microscope (BZ-X700; Keyence, Japan). All quantifications were performed at 40x magnification. Random areas (eight per suture) were analyzed, and the coverage area was estimated using the image analysis software program ImageJ (National Institute of Health, Bethesda, MD, USA). The mean coverage area of 24 images from three sutures was calculated. Further, sutures were fixed with 2.0% glutaraldehyde for 2.0 h and dehydrated in graded ethanol. The well-dehydrated suture samples were observed under a scanning electron microscopy (SEM; JCM-6000, JEOL Ltd., Tokyo, Japan) at an accelerating voltage of 15 kV after platinum deposition. The number of adherent bacteria was estimated using the field of view at 1000x magnification. Random areas (eight per suture) were recorded, and visibly adherent bacteria were counted using ImageJ. The mean number of adherent bacteria of 24 images from three sutures was calculated.

### 2.6. Evaluation of Biofilm Formation

To generate biofilms on the suture surfaces, bacterial cells were prepared to a density of 1.0 × 10^6^ bacteria/mL in TSB supplemented with 0.25% glucose. Next, the suture samples were exposed to 1.0 × 10^6^ bacterial cells in 24-well plates and incubated for 24 h at 37°C while shaking at 50 rpm. Suture samples were rinsed three times with 1.0 mL PBS and observed by fluorescence microscopy and SEM.

### 2.7. *In Vivo* Evaluation

#### 2.7.1. Animals

All experimental procedures were performed under a protocol approved by the Animal Care and Use Committee of the University of Tokyo. In brief, twelve 8-week-old male C57BL/6J mice from SLC (Shizuoka, Japan) were used in this study. All mice were housed in plastic cages with bedding material in a specific pathogen-free facility, with 12 h light-dark cycles.

#### 2.7.2. Surgical Technique

All mice were intraperitoneally injected with a mixture of anesthetic agents (0.38 mg/kg medetomidine, 2.0 mg/kg midazolam, and 2.5 mg/kg butorphanol). Hair was removed using clippers. Subcutaneous pockets were created on both sides of the back of mice, and each suture was aseptically implanted into the subcutaneous pockets: a PMB-coated suture was implanted on the right side of the mouse and a noncoated suture on the left side. MRSA suspension (5.0 × 10^8^ bacteria/mL) was inoculated onto the suture before implantation. Incisions were closed using a 5-0 nylon suture. No antibiotics were used following the operation. After 1, 3, and 7 days, the animals were euthanized, and the sutures removed. All samples were observed by fluorescence microscopy and SEM. All operations were performed by one surgeon under the same experimental conditions.

### 2.8. Statistical Analysis

All data are expressed as mean ± standard deviation. Statistical analyses were conducted with the unpaired two-tailed Student's *t*-test using BellCurve for Excel. The *p* value < 0.05 was considered significant.

## 3. Results

### 3.1. Characterization of PMB-Coated Sutures

**Figure 1:**
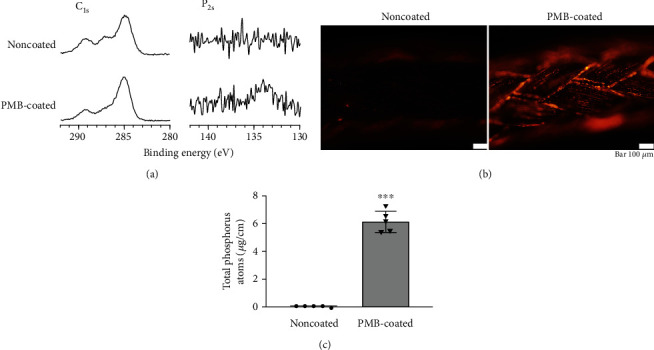
Analysis of the surface composition of biomaterials. (a) XPS spectra of sutures, C_1s_, and P_2p_. (b) Representative fluorescence micrographs of noncoated and PMB-coated sutures stained with rhodamine 6G. The scale bar indicates 100 *μ*m. (c) The absolute amount of phosphorus atoms on suture surfaces. ^∗∗∗^*p* < 0.001.

The XPS peaks of the phosphorus atom region (P_2p_), detected at 133 eV, corresponding to the phosphate group, were observed exclusively on PMB-coated sutures (Figure 1(a)). In addition, strong fluorescence was observed on the suture surface treated with PMB (Figure 1(b)), confirming that the suture was covered with sufficient amounts of PMB. The quantity of phosphorus atoms on the surface of noncoated and PMB-coated sutures was 0.09 *μ*g/cm and 6.2 *μ*g/cm (*p* < 0.001), respectively (Figure 1(c)).

PMB-coated sutures showed sustained integrity and uniformly covered the tested surfaces. There was no difference in diameter and weight between noncoated and coated sutures (Table 1). The maximum tensile forces of noncoated control and coated sutures were measured as 170 N (Table 1). The static water contact angles on the surface of noncoated and PMB-coated sutures were 38° and 31° (*p* < 0.01), respectively (Table 1).

### 3.2. Bacterial Adhesion and Growth on PMB-Coated Surfaces

**Figure 2:**
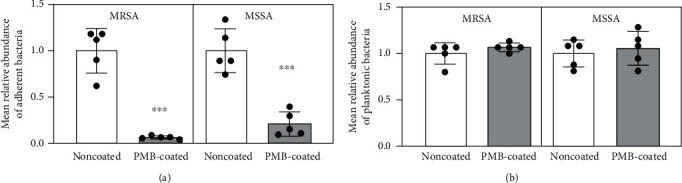
(a) Adherent and (b) planktonic bacteria on sutures after 1 h of incubation. Data are expressed as means ± standard deviation. ^∗∗∗^*p* < 0.001.

**Figure 3:**
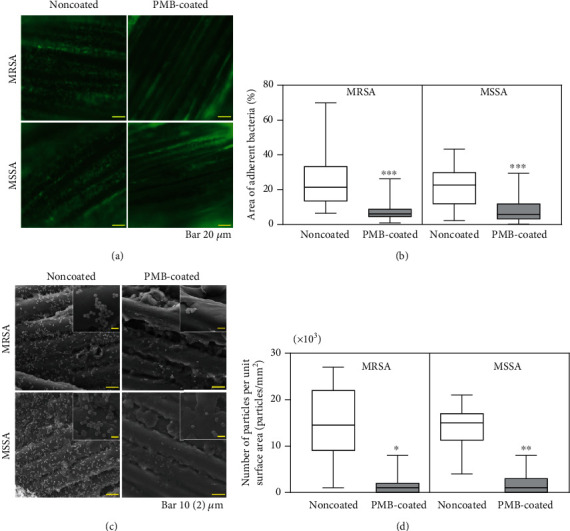
Evaluation of bacterial adhesion. (a) Representative fluorescence microscopy images of sutures after bacterial culture for 1 h *in vitro*. The scale bar indicates 20 *μ*m. (b) Box-and-whisker plots of the area of adherent bacteria on suture surfaces after bacterial culture for 1 h *in vitro*. ^∗∗∗^*p* < 0.001. (c) Representative SEM images of bacterial adherence after bacterial culture for 1 h *in vitro*. The scale bar indicates 10 *μ*m. Insets are magnified images (scale bar indicates 2 *μ*m). (d) Box-and-whisker plots of adherent bacteria on suture surfaces after bacterial culture for 1 h *in vitro*. ^∗∗^*p* < 0.01 and ^∗^*p* < 0.05.

**Figure 4:**
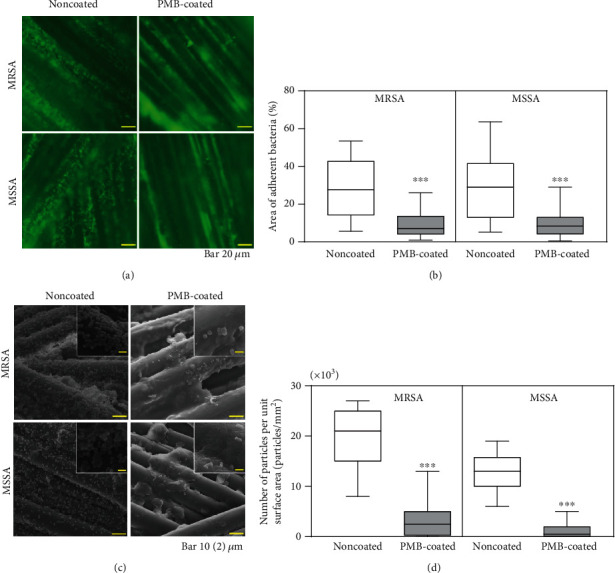
Evaluation of biofilm formation. (a) Representative fluorescence microscopy images of sutures after bacterial culture for 24 h *in vitro*. The scale bar indicates 20 *μ*m. (b) Box-and-whisker plots of the area of adherent bacteria on sutures after bacterial culture for 24 h *in vitro*. ^∗∗∗^*p* < 0.001. (c) Representative SEM images of bacterial adherence after bacterial culture for 24 h *in vitro*. The scale bar indicates 10 *μ*m. Insets are magnified images (scale bar indicates 2 *μ*m). (d) Box-and-whisker plots of adherent bacteria on suture surfaces after bacterial culture for 24 h *in vitro*. ^∗∗∗^*p* < 0.001.

The initial bacterial adhesion of MRSA and MSSA on PMB-coated suture surfaces demonstrated drastic reductions in bacterial adhesion compared to those observed on noncoated surfaces (93 and 79% reduction, respectively) (Figure 2(a)). However, there were no significant differences in the mean relative abundance of planktonic bacteria (Figure 2(b)). SYTO® 9 nucleic acid staining exhibits green fluorescence on live bacteria, which was used to measure adherent bacteria by fluorescence microscopy. The noncoated suture surface had a higher number of live bacteria than the PMB-coated suture (Figure 3(a)). Specifically, the area of adherent MRSA and MSSA was significantly lower on the PMB-coated suture (72 and 74% reduction, respectively) (Figure 3(b)). In SEM images (Figure 3(c)), similar trend of initial bacterial adhesion was observed, in which many bacterial colonies grew on uncoated suture surfaces. By contrast, very few bacterial colonies adhered to the PMB-coated suture surface (Figure 3(d)). Thus, the PMB-coated suture inhibited initial bacterial adhesion in all evaluation. For both bacterial strains, significant biofilm formation was observed on the noncoated suture. By contrast, very few biofilms were observed on the PMB-coated suture surface (Figure 4).

### 3.3. *In Vivo* Effects of PMB Coating on Biofilm Formation

**Figure 5:**
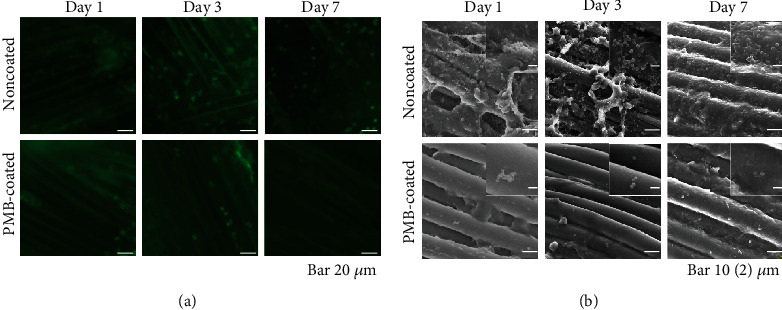
Representative images of bacterial adherence and biofilm formation after implantation for 1, 3, and 7 days *in vivo*. (a) Fluorescence microscopy images. The scale bar indicates 10 *μ*m. (b) SEM images. The scale bar indicates 10 *μ*m. Insets are magnified images (scale bar indicates 2 *μ*m).

Although biofilm formation increased over time, no mice exhibited skin disorders, poor wound healing, or died during the study. Similar to the *in vitro* findings, bacterial attachment and biofilm formation were suppressed on PMB-coated sutures (Figure 5).

## 4. Discussion

MPC contains a zwitterionic and hydrophilic phosphorylcholine group and a polymerizable methacryloyl group in its molecular structure [17, 27]. As MPC has suitable polymerization and copolymerization abilities with other vinyl monomers, through conventional radical polymerization processes, the molecular design of the MPC polymer is flexible and can be tailored to obtain desired antifouling properties. When the MPC polymer binds to a substrate by physical adsorption and adhesion, the modified surface can inhibit protein absorption and cell adhesion sufficiently [19]. Due to these beneficial properties, many medical devices have been developed using MPC polymers for clinical implementation. Since the initial adherence of microbes and adsorbed proteins to biomaterial surfaces is critical for the pathogenesis of foreign body infections [28], MPC polymers are optimal tools to prevent biofilm formation. In this study, we coated the suture surface with the MPC polymer layer to prevent suture-associated infections, which led to significant antibiofouling effects both *in vitro* and *in vivo*.

One of the MPC polymers, PMB, was designed given its solubility and thin film formation ability on the substrate. The PMB is not hydrosoluble due to its high-molecular weight and 70% hydrophobic BMA content. Due to the stable coating with PMB, in this study, we chose a 30/70-unit mole ratio of MPC/BMA. Of course, the MPC content is important to reduce bacterial adhesion. When the MPC content is smaller, the adhesion of bacteria increases. On the other hand, when the MPC content exceeds 40-unit mol%, the polymer became too hydrophilic to stably attach to the substrate. Therefore, in this and related previous studies, a 30/70-unit mole ratio of MPC/BMA simultaneously and effectively forms a coating layer and inhibits bacteria adhesion. Immediately after the coating, van der Waals forces are established between PMB and suture substrate, while hydrophobic interactions are formed upon suture contact with an aqueous medium. The molecular weight of PMB is controlled above 5 × 10^6^ to generate a stable thin film that firmly attaches to the substrate. Therefore, we used PMB to prevent SSIs based on the reduction of surface biofilm formation.

We first analyzed the surface composition of the biomaterials to determine whether PMB coating was successful. The PMB coating was confirmed by XPS, which showed the atomic detection of distinctive phosphorus and carbon peaks (Figure 1(a)). Rhodamine 6G is a cationic fluorescent dye and is known to selectively adsorb to phospholipids, which aids the visualization of phospholipid-based polymers [29]. Intense fluorescence staining was observed on the surface treated with PMB, confirming the successful coating of PMB on the whole suture surface (Figure 1(b)). Additionally, the absolute amount of phosphorus atoms on PMB-coated sutures was significantly higher than that on noncoated sutures (Figure 1(c)). From these results, we concluded that PMB properly coated the suture surfaces.

Consistent with previous studies [18, 19, 30], the water wettability of the PMB-modified suture surfaces was higher than that of noncoated sutures (Table 1), without reducing mechanical strength. Since MPC polymers have an extremely hydrophilic nature, the PMB-modified suture surface showed a hydrated layer in aqueous environments [19]. Hence, we successfully influenced bacterial adhesion and biofilm formation by treating the suture surfaces with PMB.

Expectedly, PMB-coated sutures limited bacterial adherence without affecting planktonic bacteria (Figure 2), indicating that PMB treatment has bacteriostatic but not antibacterial effects [18, 19]. In other words, PMB treatment only suppresses the initial bacterial adhesion without killing bacteria, which also prevents biofilm formation. This mechanism of action indicated that PMB poses no health-related risks.

Biofilms are microbial communities that become attached and embedded in an implanted material [31]. Biofilm formation protects bacteria against the host's immune system and antibiotics, resulting in SSIs. Here, fluorescence microscopy and SEM images revealed that PMB coating significantly inhibited bacterial adhesion and biofilm formation both *in vitro* and *in vivo* (Figures 3–5).

Our study, however, has several limitations. First, only MRSA and MSSA were examined, without considering other pertinent strains that can cause infection, including *S. epidermidis*, *Enterococcus faecalis*, and *Escherichia coli*. However, we speculate that PMB-coated sutures will be effective against all strains because the PMB-coated suture surface suppresses the first step of infection, which is protein adsorption. Second, the SYTO9 green fluorescent nucleic acid stain was used to evaluate the biofilm coverage rates. This stain reacts with both the biofilm and debris of inflammatory tissues *in vivo*. Finally, although potential resistance to acute infection was evaluated over 7 days *in vivo*, these findings do not apply to antibiofilm activity in chronic infections. Further investigations, including analysis of bacterial adhesion and complex physicochemical interactions in various environments, are needed to confirm the efficacy of PMB-coated sutures against bacterial adhesion and biofilm formation.

## 5. Conclusions

Here, we developed an antibiofouling surface by coating suture surfaces with PMB. The PMB-coated sutures exhibited mechanical strength and surface morphology equal to those of noncoated sutures. Besides, significant inhibition of MRSA and MSSA initial adhesion and biofilm formation were validated, both *in vitro* and *in vivo*. This study indicates significant bacteriostatic effects of PMB-coated sutures, implying their potential applications to reduce the risk of suture-related SSIs.

## Figures and Tables

**Table 1:** Diameter, weight, maximum tensile strength, and static water contact angle of sutures.

Suture	Diameter (mm)	Weight (mg)	Maximum tensile strength (N)	Static water contact angle (degree)
Noncoated	0.60 ± 0.01	0.36 ± 0.03	170 ± 3	38 ± 6
PMB-coated	0.61 ± 0.00	0.36 ± 0.05	170 ± 4	31 ± 5^∗∗^

Data are expressed as means ± standard deviation. ^∗∗^*p* < 0.01.

## Data Availability

The data used to support the findings of this study are included within the article.
